# Ultrasonography-guided radiofrequency ablation of vascular malformations—The moving shot technique

**DOI:** 10.3389/fmed.2023.1345904

**Published:** 2024-01-12

**Authors:** Peter B. Sporns, Marios Psychogios, Kristine Blackham, Christoph Zech, Moritz Wildgruber, Martin Takes

**Affiliations:** ^1^Department of Neuroradiology, Clinic of Radiology & Nuclear Medicine, University Hospital Basel, Basel, Switzerland; ^2^Department of Diagnostic and Interventional Neuroradiology, University Medical Center Hamburg-Eppendorf, Hamburg, Germany; ^3^Department of Radiology and Neuroradiology, Stadtspital Zürich, Zürich, Switzerland; ^4^Department of Interventional Radiology, Clinic of Radiology & Nuclear Medicine, University Hospital Basel, Basel, Switzerland; ^5^Department of Radiology, Ludwig-Maximilians-University (LMU) Munich, Munich, Germany

**Keywords:** radiofrequency ablation, moving shot technique, vascular malformation, ultrasound, arteriovenous malformation, AVM, venous malformation (VM), lymphatic malformation (LM)

## Abstract

**Purpose:**

To describe a novel ultrasound-guided technique for percutaneous radiofrequency ablation of vascular malformations—the “moving shot technique.”

**Methods:**

Preliminary observational cohort study, conducted from June 1, 2019, to January 31, 2021, including all consecutive patients diagnosed with vascular malformations who were treated with ultrasound-guided radiofrequency ablation using the moving shot technique. Only patients who had undergone at least one unsuccessful previous treatment were included (sclerotherapy with ethanol/aethoxysklerol or embolization/surgery).

**Results:**

Eight patients with a median age of 22 years (interquartile range, 13–31) were included. Patients had different vascular malformations consisting of 1 arteriovenous malformation, 4 venous malformations, and 1 each a mixed venous-lymphatic malformation, a glomuvenous malformation and a FAVA (fibroadipose vascular anomaly). Malformations were located at the limbs in 5 patients (62.5%), the subcutaneous/intramuscular tissue of the body in 2 patients (25%) and at the chin in 1 patient (12.5%). Clinical symptoms were pain in 8 patients (100%), swelling in 6 patients (75%), and partial immobility in 4 patients (50%). All patients showed an improvement of clinical symptoms after treatment with 7 (87.5%) being completely asymptomatic and 1 (12.5%) showing improvement of immobility and pain. No procedural complications, such as nerve damage or skin burns occurred.

**Conclusion:**

The moving shot technique using ultrasonography-guided radiofrequency ablation is a promising technique for the interventional treatment of vascular malformations and should be validated in multicenter-approaches.

## Introduction

Vascular malformations are abnormalities in blood vessel morphogenesis with an overall prevalence of approximately 1.2–1.5% (1, 2). According to the classification of the International Society for the Study of Vascular Anomalies (ISSVA) (3–5) vascular malformations are divided in slow-flow and high-flow lesions, related to the flow pattern, which is crucial both for treatment decision and the prognosis (6). Slow flow malformations include venous malformation (VM), lymphatic malformation (LM), or arteriovenous malformation (AVM). Slow-flow malformations represent the majority (>90%) of vascular malformations and among these, venous malformations (VMs) are the most common type (2).

For the treatment of vascular malformations, several interventional therapies such as percutaneous sclerotherapy or embolization have been reported, mostly depending on the flow pattern (7, 8). Especially lesions involving the skin or adjacent to neurovascular structures are prone to major procedural complications such as soft tissue necrosis or nerve palsy (9). Ultrasonography (US)-guided radiofrequency ablation (RFA) has been used to treat various types of benign and malignant tumors in many organs including hepatocellular carcinomas, hepatic metastases, and thyroid nodules (10–12). One recent study described the use of US-RFA for venous malformations and found a good safety profile. yin The combination with the moving shot technique, which is established for RFA of thyroid nodules (11, 12) may enable a complete treatment of malformations with a low complication rate. Hence, we report our experience with this new technique and hypothesized that it can be used for effective therapy of different types of vascular malformations safely.

## Materials and methods

### Patients

This preliminary observational cohort study, conducted from June 1, 2019, to January 31, 2021, included all consecutive patients diagnosed with vascular malformations who were treated with US-RFA using the moving shot technique. All patients had undergone at least one unsuccessful previous treatment (sclerotherapy with ethanol/aethoxysklerol or embolization/surgery). The study was approved by the local ethics committee in accordance with the Declaration of Helsinki, with waiver of informed consent.

Vascular malformation were classified according to the classification of the International Society for the Study of Vascular Anomalies (ISSVA) (3). Further, AVMs were classified using the Schobinger classification as a clinical assessment of vascular shunting (13) and the Cho classification describing angiographic characteristics (14). Venous malformations were classified using the Puig classification containing information on venous drainage patterns (15).

### Outcomes measures

Clinical outcome parameters included visual analogue scales (VAS) to measure pain severity before and after treatment as well as assessment of movement of limbs at admission and post-interventional stage. This data were collected by the treating interventional radiologist, when seeing the patient for pre-interventional and post-interventional clinics. CIRSE classification system for complications was applied to measure per-interventional complications (16). The volume calculations of the malformations in admission and follow-up imaging were performed using a three-dimensional semi-automatic technique with an established open-source software (Medical Imaging Toolkit; MITK, Istituto di Calcolo ad Alte Prestazioni, Napoli, Italy) (17, 18).

### Equipment and technique

We used an 18-gauge, monopolar, modified, internally cooled electrode with a 0.7–1.5 cm active tip and a 7–10 cm shaft length, specifically developed for thyroid lesions (11). A peristaltic pump (all HS AMICA, Spain) continuously infused cold saline (0°C) into the lumen of the electrodes to maintain the temperature of the electrode. Ablation was performed using 20–70 watts in pulsing mode. The electrode tip was initially positioned under US-guidance at a deep part of the malformation. The electrode tip moves slowly but at continuous speed in order to prevent the RFA cut-off phenomenon. When an echogenic area was detected at the tip of the electrode, it was repositioned looking for malformative vessels under US-guidance in the Duplex mode. The RFA starts at the parts further away from the US-transducer and moves to the closer parts because gas from the denaturation may reduce image quality. Treatment success was demonstrated by US and by controlling the temperature of the tissue around the needle which should be >50°C for 5 min or once >60°C (19).

### Statistical analysis

Univariable distribution of metric variables is described by median and interquartile range. For categorial data, absolute and relative frequencies are given. Due to small sample size only descriptive statistics were used. All statistical analyses were performed using SPSS, version 22 (IBM Software, Chicago, IL, USA).

## Results

### Patients

Overall, eight patients were included; five (62.5%) were male, and three (37.5%) were female. Median age was 22 years [total range, 11–56; interquartile range (IQR), 13–31]. One patient had an arteriovenous malformation (classified as Schobinger II and Cho IIIb), four had venous malformations (2x Puig type I, 1x type II and 1x type III), and one each had a mixed venous-lymphatic malformation, a glomuvenous malformation and a FAVA (fibroadipose vascular anomaly). Malformations were located at the limbs in 5 patients (62.5%), the body in 2 patients (25%) and at the chin in 1 patient (12.5%). Clinical symptoms were pain in eight patients (100%, median VAS 5), swelling in six patients (75%), and partial immobility in four patients (50%, 4 of 5 patients with limb malformations). Median time of clinical follow-up was 6 weeks (IQR 6–25) (Table 1). Patients who were asymptomatic at 6-week follow-up were not further followed.

**TABLE 1:** Baseline characteristics of patients.

	All patients (*n* = 8)
**Demographics**	
Age, median (IQR), year	22 (13–31)
Women, *n* (%)	3 (37.5)
**Malformation**	
AVM, *n* (%)	1 (12.5)
Venous malformation, *n* (%)	4 (50)
Venous-lymphatic malformation, *n* (%)	1 (12.5)
Glomuvenous malformation, *n* (%)	1 (12.5)
FAVA	1 (12.5)
**Location of malformation**	
Body subcutaneous/intramuscular, *n* (%)	2 (25)
Limbs, *n* (%)	5 (62.5)
Chin, *n* (%)	1 (12.5)
**Symptoms before treatment**	
Pain, *n* (%)	8 (100)
Swelling, *n* (%)	6 (75)
Immobilization, *n* (%)	4 (50)
Median visual analog scale, VAS (range)	5 (2–9)
Previous therapy (%)	8 (100)
Aethoxysklerol, *n* (%)	6 (87.5)
Alcohol, *n* (%)	1 (12.5)
Embolization/operation	1 (12.5)
Time of clinical follow-up, median (IQR), weeks	6 (6–25)
**Modality of follow-up**	
Ultrasound	5 (62.5)
Additional MRI	3 (37.5)
Volume reduction, mean (%), IQR	96.25 (90–100)
Complications, *n* (%)	0 (0)
**Clinical change**	
Asymptomatic, *n* (%)	7 (87.5)
Improvement, *n* (%)	1 (12.5)
Worsening, *n* (%)	0 (0)
Median visual analog scale, VAS (range)	0 (0–1)

FAVA, fibro-adipose vascular anomaly; AVM, arteriovenous malformation; IQR, interquartile range.

### Outcome and safety

All patients showed an improvement of clinical symptoms after treatment with seven (87.5%) being completely asymptomatic and one (12.5%) showing improvement of symptoms and just slight remaining pain. Median VAS for quantification of pain decreased from 5 (range 2–9) at admission to 0 (range 0–1) at follow-up. All patients could move normally at follow-up with no residual immobility.

All patients underwent imaging to determine the degree of volume reduction of the malformation; five (62.5%) had ultrasound and three (37.5%) had additional magnetic resonance imaging. Mean volume of the malformation at admission was 76 cm^3^ and mean reduction was 96% (to a mean of 3 cm^3^, IQR 90–100). No procedural complications, such as nerve damage or skin burns occurred. There was no deviation from the normal post-therapeutic course (according to CIRSE classification system for complications) (16).

## Discussion

Our study describes the moving shot technique using US-RFA in patients with different types of vascular malformations. In our preliminary experience the mean reduction of the volume of the treated malformations was 96%, clinical symptoms improved in all patients (with 87% being completely asymptomatic) and a good safety profile.

Whereas a recent study described a similar approach for the treatment of venous malformation, our study also included other types of vascular malformations such as AVMs, and thereby provides first evidence that a broad spectrum of malformations may be treated using this technique.

For venous malformations, besides conservative management, invasive therapy is indicated in symptomatic VMs to reduce symptoms such as pain, hemorrhage, and impairment of neighboring structures. For VMs percutaneous sclerotherapy is the first-choice invasive treatment method and can be combined with laser therapy or surgical procedures (20, 21). However, evidence is low and the choice for the invasive method remains a shared decision between the patient and a multidisciplinary team of specialists (22, 23). Likewise symptomatic macrocystic lymphatic malformations can effectively be treated using percutaneous sclerotherapy with a good safety profile (24), whereas for microcystic LMs percutaneous sclerotherapy may be less effective and requires other sclerosants such as Picibanil, Bleomycin, and Doxycycline (8).

For high-flow vascular malformations such as AVMs invasive therapy is indicated in patients with progressive symptoms according to the Schobinger classification. Here, embolization is the first choice with a relatively low morbidity but may be prone to recurrence (20, 25).

The main advantage of RFA compared to other embolization techniques or sclerotherapies is that the vessels of the malformation are completely ablated and no material such as onyx remains, which may lead a complete remodeling of the tissue and thereby to a larger proportion of patients with clinical improvement. Moreover, the nidus may sometimes be difficult to reach using embolization techniques. Therefore, US-RFA offers another, technically completely different approach, which may lead to lower recurrence rates in cases where embolization therapy is difficult due to anatomical reasons or the arterial supply of the AVM (26).

Our study has limitations such as the retrospective single-center design and the small sample size. Moreover, imaging follow-up was not homogenous as only three patients underwent additional MRI.

## Conclusion

The moving shot technique using ultrasound-guided radiofrequency ablation is a promising technique for the interventional treatment of different types of vascular malformations.

## Data availability statement

The original contributions presented in the study are included in the article/supplementary material, further inquiries can be directed to the corresponding author.

## Ethics statement

The studies involving humans were approved by the Ethics committee of University of Basel. The studies were conducted in accordance with the local legislation and institutional requirements. The Ethics Committee/Institutional Review Board waived the requirement of written informed consent for participation from the participants or the participants’ legal guardians/next of kin because this was a retrospective analysis.

## Author contributions

PS: Conceptualization, Data curation, Formal analysis, Funding acquisition, Investigation, Methodology, Project administration, Resources, Supervision, Validation, Visualization, Writing – original draft, Writing – review and editing. MP: Funding acquisition, Resources, Supervision, Validation, Writing – review and editing. KB: Supervision, Validation, Writing – review and editing. CZ: Supervision, Validation, Writing – review and editing. MW: Conceptualization, Data curation, Formal analysis, Investigation, Methodology, Project administration, Resources, Supervision, Validation, Visualization, Writing – original draft, Writing – review and editing. MT: Conceptualization, Data curation, Formal analysis, Investigation, Methodology, Resources, Supervision, Validation, Visualization, Writing – original draft, Writing – review and editing.
